# Detecting Heterogeneity of Intervention Effects Using Analysis and Meta-analysis of Differences in Variance Between Trial Arms

**DOI:** 10.1097/EDE.0000000000001401

**Published:** 2021-08-20

**Authors:** Harriet L. Mills, Julian P.T. Higgins, Richard W. Morris, David Kessler, Jon Heron, Nicola Wiles, George Davey Smith, Kate Tilling

**Affiliations:** From the a Medical Research Council Integrative Epidemiology Unit, Bristol Medical School, University of Bristol, Bristol, United Kingdom; b Population Health Sciences, Bristol Medical School, University of Bristol, Bristol, United Kingdom; c National Institute for Health Research Bristol Biomedical Research Centre, University Hospitals Bristol NHS Foundation Trust and University of Bristol, Bristol, United Kingdom.

**Keywords:** Effect modification, Heterogeneity, Meta-analyses, Randomized controlled trials, Subgroups, Subgroup analysis, Variance

## Abstract

Randomized controlled trials (RCTs) with continuous outcomes usually only examine mean differences in response between trial arms. If the intervention has heterogeneous effects, then outcome variances will also differ between arms. Power of an individual trial to assess heterogeneity is lower than the power to detect the same size of main effect.

**Methods:**

We describe several methods for assessing differences in variance in trial arms and apply them to a single trial with individual patient data and to meta-analyses using summary data. Where individual data are available, we use regression-based methods to examine the effects of covariates on variation. We present an additional method to meta-analyze differences in variances with summary data.

**Results:**

In the single trial, there was agreement between methods, and the difference in variance was largely due to differences in prevalence of depression at baseline. In two meta-analyses, most individual trials did not show strong evidence of a difference in variance between arms, with wide confidence intervals. However, both meta-analyses showed evidence of greater variance in the control arm, and in one example, this was perhaps because mean outcome in the control arm was higher.

**Conclusions:**

Using meta-analysis, we overcame low power of individual trials to examine differences in variance using meta-analysis. Evidence of differences in variance should be followed up to identify potential effect modifiers and explore other possible causes such as varying compliance.

## Abstract 2

Supplemental Digital Content is available in the text.

## INTRODUCTION

In medical research, we often estimate the average effect of an intervention by comparing the mean outcome between arms in a randomized controlled trial (RCT). However, individual responses to interventions may vary—the effectiveness of an intervention might decrease with age, or there might be subgroups for whom the intervention has no effect. In the era of personalized (or stratified) medicine, there is increasing interest in identifying these effect modifiers or subgroups.^1^

Here, we focus on trials with a continuous outcome, where the main effect is the mean difference between two arms of a trial. Identification of effect modifiers or subgroups is often approached by testing for statistical interactions. A potential effect modifier is specified (usually a priori, for RCTs) and the null hypothesis is that the effect of the intervention on the outcome does not vary over the levels of the modifier (i.e., that there is no additive interaction). A trial powered to detect such an interaction needs to be approximately four times the size of a trial powered to detect a similar magnitude of overall treatment effect,^2,3^ or larger if the subgroups are very different in size. Multiple testing can be a problem if interactions with many covariates are examined, with a risk of overfitting,^4^ although this can be minimized by using model selection methods.^5–8^ All these methods require knowledge of, and data on, the potential effect modifiers: if an effect modifier is not measured, then its interaction with the intervention cannot be tested. An alternative way to investigate effect modification, without prespecifying the effect modifiers, is to examine whether variance in the outcome differs between the arms of the trial.^9–12^ If the effect of the intervention is the same for all participants then the variation of the outcome in the intervention arm will be the same as the that in the control arm. However, if the effect of the intervention varies across participants, then the variation of the outcome in the intervention arm will be different to that in the control.^9,13,14^ A difference in variance would then require further study to identify the effect modifiers, using individual participant data.

As with the test for a specific effect modifier, power to detect a difference in variances will be low in a single trial powered to detect a difference in means. However, meta-analysis of differences in variance should give increased power to detect effect modification. A small number of meta-analyses in epidemiology and ecology have reported on differences in variance,^9,10,15–23^ with applications to RCTs and other types of comparative study. Most of them found evidence of a difference in variance between arms, with varying strength of evidence (eTable 1; http://links.lww.com/EDE/B835).

Here, we describe and implement methods for examining the effect of an intervention on the variance of an outcome, both in a single trial (with individual participant data) and using meta-analysis to combine across trials (using summary data). We describe the assumptions behind each method and show how to conduct further analyses with individual participant data to investigate which variables might be causing the effect modification. We use simulations to show that decisions about when to examine the association between overall mean and variance should not be based on reported means and variances from individual trials. We then illustrate the methods using individual participant data from an RCT of cognitive behavioral therapy (CBT) to treat depression, and summary data from meta-analyses of RCTs examining the effect of (1) computer-based psychological treatments on depression and (2) statins on low-density lipoprotein (LDL) cholesterol.

## METHODS FOR EXAMINING DIFFERENCE IN VARIANCE BETWEEN TRIAL ARMS

Examining differences in variance between two arms using data from one trial.

We review methods briefly here, presenting more detail in Table 1 and formulae in eAppendix 2; http://links.lww.com/EDE/B835.

**TABLE 1.** Methods for Examining Differences in Variance Between Two Arms, and for Examining the Relationship Between Mean and Variation Across the Two Arms

Test Name	Description	Minimum Requirements and Assumptions
*Testing differences in variance between two arms using data from one trial*		
Glejser test^24^	The absolute values of the residuals from a standard linear model of outcome against treatment are regressed on the treatment indicator	Requires individual participant data (IPD)
		Assumes normality
		Can include covariates
		Can be defined for k > 2 arms
Levene test^25^	Levene’s test statistic has approximate F-distribution with 1 and N − 2 degrees of freedom	Requires IPD
		Suitable for nonnormal data
		Can be defined for k > 2 arms
		Can be defined using the mean, trimmed mean or median (the Brown-Forsythe test^49^)
Bartlett test^26^	Bartlett’s test statistic has approximate chi-squared distribution (1 degree of freedom) when variances are equal	Can be calculated using IPD or summary data (sample sizes, SDs)
		Assumes normality
		Can be defined for k>2 arms
*Estimating differences in variance between two arms using data from one trial*		
Linear model with nonconstant variance	A linear model that assumes a different residual variation in each arm.	Requires IPD
		Assumes normality
		Can include covariates
		Can be defined for k > 2 arms
Difference in variances	The difference in sample variances and its standard error are used to calculate a test statistic with an approximate normal distribution, so a t-test is used to compare variances	Can be calculated using IPD or summary data (sample sizes, SDs)
		Assumes normality
Ratio of variances, F-test	The ratio of sample variances between the two arms has approximate F-distribution with N0 −1 and N1 −1 degrees of freedom, if the true variances are equal	Can be calculated using IPD or summary data (SDs)
		Assumes normality
Log of the ratio of SDs^9,27 a^	The log of the ratio of SDs and the sampling variance are used to calculate a test statistic with approximate normal distribution, so a t-test is used to compare variances	Can be calculated using IPD or summary data (sample sizes, SDs)
		Assumes normality
*Examining the relationship between mean and variance across the two arms*		
Difference in coefficient of variation^29^	The difference in CoVs and its standard error are used to calculate a test statistic, whose square has approximate chi-squared distribution (1 degree of freedom)	Can be calculated using IPD or summary data (sample sizes, SDs, means)
		Assumes normality
		Data must be on a ratio scale with a meaningful zero
		This test performs best if each >10 and each >0.33.^29^
Log of the ratio of coefficients of variation^27^	The log of the ratio of CoVs and the sampling variance are used to calculate a test statistic with approximate normal distribution, so a t-test is used to compare arms	Can be calculated using IPD or summary data (sample sizes, SDs, means)
		Can be made suitable for nonnormal data by additions to the equation for sample variance
		Data must be on a ratio scale with a meaningful zero

Further method details (and equations) are in eAppendix 2. Code for each method in R is provided online (https://github.com/harrietlmills/DetectingDifferencesInVariance).

^a^Note that this is called log of the variability ratio, logVR in these two references.

The null hypothesis of equal variances in both arms can be tested using Glejser,^24^ Levene,^25^ or Bartlett test.^26^ The difference in variances and its standard error can be estimated either using a linear model with nonconstant variance, or using summary data, as we propose here. Finally, the ratio of the SDs or the log of the ratio of SDs (logRoSD,^9,27^ also sometimes called the log of the variability ratio) can be estimated, together with their standard errors.

We implemented all methods and analyses in R^28^ and code is available online (https://github.com/harrietlmills/DetectingDifferencesInVariance).

### Examining the Relationship Between Mean and Variation Across the Two Arms

If the mean is related to the variance for an outcome, then a homogeneous treatment effect could lead to a difference in variance between the two arms of the trial. The coefficient of variation (CoV) is the ratio of the SD to the mean: comparing CoVs between two arms will identify whether the SD differs more, or less, between the two than would be predicted by the difference in means.

We describe two methods using CoV: a difference in CoVs^29^ and the log of the ratio of CoVs (log ratio of CoV^27^), Table 1; http://links.lww.com/EDE/B835, and eAppendix 2; http://links.lww.com/EDE/B835.

CoV should only be used when the outcome data are on the ratio scale, that is, the scale has a clear definition of 0 and the ratio of two values has a meaningful interpretation. The CoV assumes that the SD is directly proportional to the mean. Therefore, it is only relevant for variables for which a sample mean of zero would imply a sample SD of zero. A variable for which CoV would be appropriate is serum cholesterol, which is measured on the ratio scale (a value of 6 is twice a value of 3), and has a meaningful zero (the value 0 mg/dL indicates that there is no measurable cholesterol in 1 dL of blood). A sample with a mean serum cholesterol of zero indicates that all the values must be zero (as serum cholesterol cannot be negative), and therefore that the sample SD must be zero. CoV has been used with outcomes which do not satisfy these criteria, for example, the Hamilton Depression Rating Scale, or the Montgomery–Asberg Depression Rating Scale, which are both interval (not ratio) scales.^17,20^

### Comparison of Methods

The linear model with nonconstant variance method and Glejser test can incorporate covariates (which may be continuous or categorical), to examine whether the heterogeneity in outcome between the arms of the trial is explained by the covariates. The linear model with nonconstant variance method and Glejser, Levene, and Bartlett tests can be defined for multiple (k>2) arms. Bartlett’s test, difference in variances, ratio of variances, log of the ratio of SDs, difference in CoV and log of the ratio of CoV can be calculated using only standard summary data (sample sizes, means, and SDs).

All tests except Levene assume data are normally distributed: if data are normally distributed Levene’s test would be expected to have lower power. All the other tests are sensitive to non-normality of the outcome, for example if the subgroups have caused a bimodal distribution, then differing responses have caused skew. Normality usually cannot be verified when only summary data are available (although evidence against normality, e.g., asymmetry of distributions, may be available by comparing mean and median).

### Methods for Use with Summary Data from Meta-analyses

The approach to meta-analysis will depend on whether the result obtained from each trial is a statistical test or an estimate. In general, we favor estimation, preferring estimates of differences in variance, ratio of variances, log ratio of SD and comparisons of CoVs (differences in CoV and log ratio of CoV). Estimates that are accompanied by standard errors can readily be meta-analyzed using standard methods (here, the difference in variance and difference in CoV methods). Ratio of variances, log ratio of variances, and log ratio of CoV can be meta-analyzed using bespoke methods using a random-effects model with restricted maximum likelihood estimates of the ratios (ratio of variances^21^; log ratio of SD, and log ratio of CoV^9,27^). If variances within arms are very different between trials in a meta-analysis, ratio methods may be preferable.

Although not covered here, synthesis of findings from statistical tests from individual trials (e.g., Bartlett test and the F-test based on the ratio of variances) could be undertaken using meta-analysis of *P* values. These produce a global *P* value to test the null hypothesis, although it can be difficult to determine whether failure to reject the null is due to small differences in variance or to an insufficient amount of evidence.

Previous analyses have implied that CoV should only be explored in a meta-analysis if the SDs and means within each trial arm are correlated.^9,17^ By simulating trial data (eAppendix 3; http://links.lww.com/EDE/B835), with (A) same CoV and (B) different CoV in the arms, we have shown that the correlation of the mean and SD from individual trials is not necessarily indicative of the CoV or whether the CoV differs between arms of the trial (eFigures 1 and 2; http://links.lww.com/EDE/B835). Thus, CoV should be used only if the outcome is a ratio variable with a true zero, irrespective of the observed correlation between SDs and means within trial arms.

## APPLIED EXAMPLES

### Analysis of a Single Trial

We first apply the methods to individual participant data from a trial of therapist-delivered internet psychotherapy for depression in primary care,^30^ chosen because the original trial report had evidence of effect modification (the intervention had a greater effect in participants with more severe depression at baseline than in those with mild depression). This RCT randomly assigned 297 individuals to either usual care while on a waiting list for CBT (control) or usual care in addition to online CBT delivered by a therapist (intervention).^30^ Baseline depression was measured using the Beck Depression Inventory (BDI)^31,32^; individuals recruited to the trial had to have a BDI score of 14 or more. BDI is a self-report questionnaire with 21 statements that patients rank from 0 to 3 (i.e., total scores are integer and in the range 0–63), with a higher score indicating more severe depression.^31,32^ We investigated BDI at 4 months as a quantitative outcome. Equality of variances between the control and intervention arms was tested using: (1) linear model with nonconstant variance (with and without adjusting for covariates); (2) Glejser test (with and without adjusting for covariates); (3) Levene test (using deviation from the mean, median and trimmed mean); (4) Bartlett test; (5) difference in variances; (6) ratio of variances (F-test) method; and (7) log ratio of SD method. The difference in CoV and log ratio of covariance methods were not included as BDI is not a ratio scale.

To examine the impact of differential dropout, we also tested the equality of variances between the control and intervention arms at baseline for (1) everyone and (2) the subset of those remaining after excluding individuals lost to follow up at 4 months, using the Bartlett, Levene, and F tests.

### Meta-analyses

We applied the summary data methods to two meta-analyses: (1) a meta-analysis of 19 RCTs of computer-based psychological treatments for depression,^33^ chosen because it included the single trial we assess above and (2) a Cochrane Review examining HMG CoA reductase inhibitors (statins) for people with chronic kidney disease,^34^ chosen because there is evidence that some people may respond to statins better than others.^35^ These meta-analyses were suitable examples because the data were appropriate for the tests we wanted to demonstrate, and the data were given in enough detail (i.e., mean, SD, and N given across both arms for all trials).

For the first meta-analysis,^33^ the outcomes were self-reported measures of depression, including the BDI. The meta-analysis found a pooled standardized mean difference of d = −0.56 (95% confidence interval [CI] −0.71, −0.41) for self-reported depression post treatment (using a random effects model),^33^ supporting the efficacy of computer-based treatments. We selected only those trials which measured BDI (or derivatives of BDI) and analyzed only one posttreatment effect per trial. We meta-analyzed the difference in variances, ratio of variances and log ratio of SD across trials, but did not include the difference in CoV or log ratio of CoV methods as BDI is not a ratio scale measure.

The second set of meta-analysis data are from analysis 1.14 in the Cochrane Review, for 22 trials reporting the effect of statins versus placebo or no treatment on LDL cholesterol (mg/dL).^34^ The meta-analysis found a pooled mean difference of −44 mg/dL (95% CI −54, −34), for the effect of statins on LDL cholesterol,^34^ confirming that statins lower serum LDL cholesterol. LDL cholesterol is measured on a ratio scale, with a meaningful zero, and thus, we meta-analyzed the difference in variances, ratio of variances, log ratio of SD, difference in CoV, and log ratio of CoV across trials.

## RESULTS

### Analysis of a Single Trial

Of the 297 individuals recruited to the trial at baseline, 210 completed 4-month follow-up (113 in the intervention arm and 97 in the control arm, Table 2).^30^ The BDI score had decreased in both arms, with a larger magnitude decrease in the intervention arm. The BDI scores were normally distributed at baseline, but not at the 4-month follow-up (eFigure 3; http://links.lww.com/EDE/B835).

**TABLE 2.** The Baseline BDI Score and Outcome BDI Score at 4 Months from the Trial Described in Kessler 2009^30^

	Group 1 (Intervention)			Group 2 (Control)		
Timepoint	N	Mean	SD	N	Mean	SD
Baseline	149	32.8	8.3	148	33.5	9.3
4 months	113	14.5	11.2	97	22.0	13.5

Table 3 shows the results of all tests of the equality of variance of BDI at 4 months. Although the data at 4 months were not normally distributed, the conclusions from all tests were similar to the Levene test, with the *P* values for all but adjusted model 1 being between 0.05 and 0.07, giving weak evidence of lower variance in the intervention arm of the trial (estimated difference in variance −57 [95% CI −118, 4.1)]. The variance is lower in the intervention arm, indicating that the treatment tends to bring participant’s BDI scores closer together; that is, the treatment tends to work best for those with high BDI scores (in line with the conclusions of the trial^30^). Including baseline BDI score (adjusted model 2 using a linear model with nonconstant variance [LMNCV] and Glejser test) attenuated the difference in variance between the arms (−21 [95% CI −191, −2.3]).

**TABLE 3.** Tests for Difference in Variance in BDI Score at 4 Months, Between the Intervention and Control Arms from the Single Trial Exploring the Effect of a CBT Intervention on Depression^30^

Test	Test Statistic		***P***	Estimate	95% CI
Unadjusted LMNCV^a^	Chi-square statistic (df = 1)	3.66	0.056	−56	(−163, −19)
Adjusted LMNCV 1^a,b^	Chi-square statistic (df = 1)	4.62	0.032	−62	(−162, −24)
Adjusted LMNCV 2^a,c^	Chi-square statistic (df = 1)	0.83	0.360	−21	(−191, −2.3)
Glejser test, unadjusted^a^	t-statistic	1.97	0.050	NA	NA
Glejser test, adjusted 1^a,b^	t-statistic	2.10	0.037	NA	NA
Glejser test, adjusted 2^a,c^	t-statistic	0.80	0.420	NA	NA
Levene test (median)	F-statistic (df = 1 and 208)	3.52	0.062	NA	NA
Levene test (mean)	F-statistic (df = 1 and 208)	3.89	0.050	NA	NA
Levene test (trimmed mean)	F-statistic (df = 1 and 208)	3.63	0.058	NA	NA
Bartlett test^a^	Chi-square statistic	3.63	0.057	NA	NA
Difference in variances	t-statistic	−1.90	0.057	−57	(−118, 4.1)
Ratio of Variances: F-test^a^	F-statistic (df = 112 and 96)	0.69	0.056	0.69	(0.47, 1.0)^d^
Log of the ratio of SDs	t-statistic	−1.92	0.056	−0.19	(−038, 0.004)

^a^The 4-month data were not normally distributed, so all tests except Levene test may have reduced power and/or bias. Also note the standard error for these estimates in the LMNCV method was obtained from Stata, replicating the analysis in R.

^b^Covariates added in the adjusted LMNCV are as specified in the original trial paper: center ID, present antidepressant treatment, sex, whether or not GP practice has a counselor.

^c^As adjusted LMNCV 1, but also including baseline BDI score.

^d^CI was derived using an F-distribution.

The analysis of baseline variances showed no differences between the two arms at baseline, even when restricting to only those with follow-up data at 4 months (eAppendix 4.2 and eTable 5; http://links.lww.com/EDE/B835).

### Meta-analyses

Our simulations confirmed that power to detect heterogeneity in single trials was low unless the trial was very large (see eAppendix 6; http://links.lww.com/EDE/B835). Therefore, we next examined the methods within a meta-analysis setting.

**FIGURE 1.**
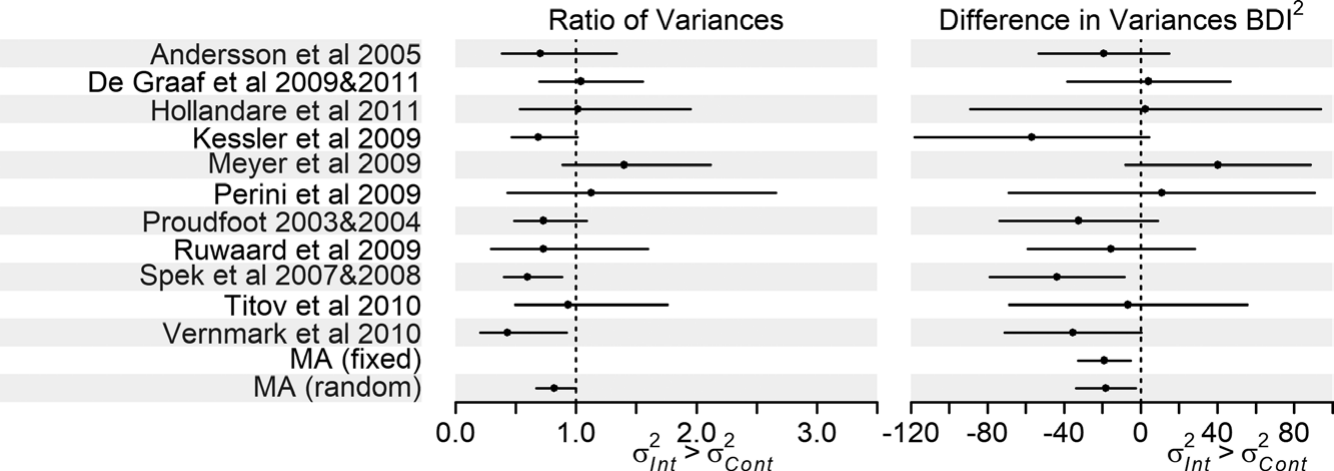
Forest plot of the ratio of variances and differences in variance analyses for the trials in the Richards et al. meta-analysis on computer-based psychological treatments for depression,^33^ results in eTable 6; http://links.lww.com/EDE/B835 (note we do not plot the results of the log ratio of SD analysis as trends are the same as the ratio of variances analysis). Please note that the studies named in the figure are those in the Richards et al. meta-analysis,^33^ and full information on these studies, including references, can be found in that article.

We restricted the meta-analysis on computer-based psychological treatments for depression^33^ to the 11 trials reporting BDI outcomes, varying in size from 44 to 216 participants. There was evidence of greater variance in the control arm (Figure 1, eTable 6; http://links.lww.com/EDE/B835: ratio of variances 0.82 [95% CI 0.67, 1.00]; difference in variances fixed-effects estimate −19 [95% CI −33, −5.5], random-effects estimate −18 [95% CI −34, −2.6]). Using the log of the ratio of SDs gave the same trends as the ratio of variances test, eTable 6; http://links.lww.com/EDE/B835. There was no strong evidence of heterogeneity in the difference in variances meta-analysis (I^2^ = 20%). Most of the individual trials showed evidence of greater variance in the control arm but CIs were wide (Figure 1, eTable 6; http://links.lww.com/EDE/B835).

The 22 trials in the meta-analysis reporting the effect of statins versus placebo or no treatment on LDL cholesterol,^34^ varied in size from 199 to 374 total participants.

**FIGURE 2.**
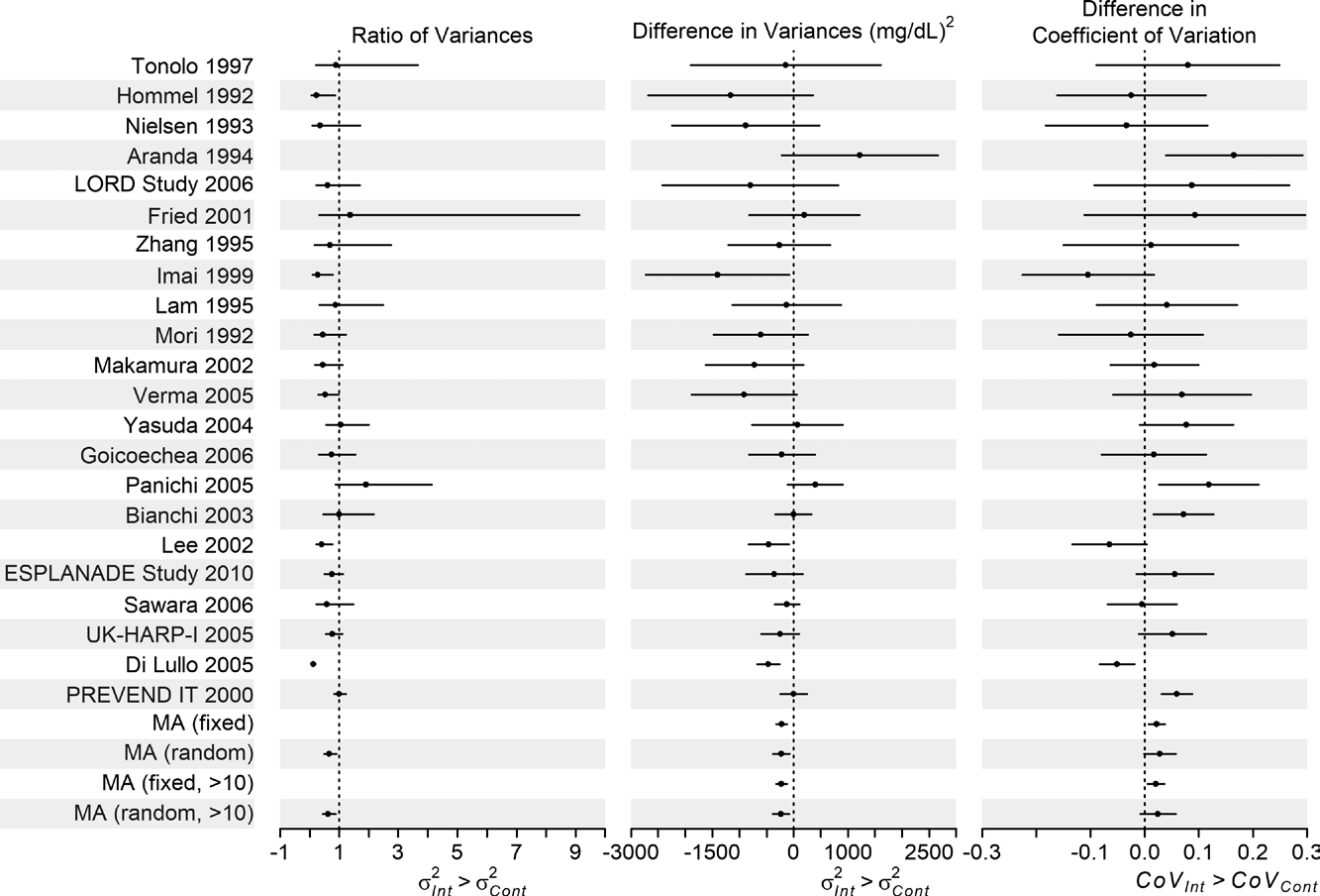
Forest plot of the ratio of variances, differences in variance, and differences in covariance analyses of the trials in the Palmer et al. meta-analysis reporting the effect of statins versus placebo or no treatment on LDL cholesterol,^34^ results in eTable 7; http://links.lww.com/EDE/B835. We have not plotted the ratio of variances results for Aranda 1994 as the ratio of variances for this trial is on a much larger scale than the others (9.51 [95% CI 1.90, 47.49]); however, it is included in the overall analysis. Note we do not plot the results of the log ratios of SDs or log ratios of covariance analyses as trends were the same as the ratio of variances and differences in covariance analyses, respectively (eTable 7; http://links.lww.com/EDE/B835). Please note that the studies named in the figure are those in the Palmer et al. meta-analysis,^34^ and full information on these studies, including references, can be found in that paper.

The meta-analysis of differences in variance showed evidence of greater variance in LDL cholesterol in the control arm (fixed-effect estimate −220 [95% CI −319, −122] mg^2^/dL^2^, random-effects estimate −226 [95% CI −377, −76] mg^2^/dL^2^). Conclusions were unchanged when only trials with more than 10 cases in both arms were included (excluding six trials). The pooled ratios of variance also showed evidence of greater variance in the control arm 0.66 [95% CI 0.48, 0.91] (Figure 2, eTable 7; http://links.lww.com/EDE/B835). Conclusions were unchanged if the six smallest trials were excluded. Trends for the logRoSD analysis were the same as the ratios of variance analysis, eTable 7; http://links.lww.com/EDE/B835. There was no strong evidence of heterogeneity in the DiV meta-analysis (I^2^ = 36.5%). Most individual trials showed evidence of greater variance in the control arm, but CIs were wide (Figure 2, eTable 7; http://links.lww.com/EDE/B835).

There was weak evidence of a difference in coefficient of variation (DiCV) between arms (Figure 2, eTable 7; http://links.lww.com/EDE/B835: 0.02 [95% CI 0.01, 0.03] for fixed effects, and 0.03 [95% CI: −0.00, 0.06] for a random-effects model. Conclusions were unchanged with the six smallest trials excluded. Trends for the log of the ratio of CoV analysis were the same (eTable 7; http://links.lww.com/EDE/B835). The CoV is larger in the intervention arm than in the control arm, that is, the SD is a larger multiple of the mean in the intervention than the control arm. This suggests weak evidence of more variation in the intervention arm than would be expected given the difference in means (also reflected in individual trial results, Figure 2 and eTable 7; http://links.lww.com/EDE/B835), which could be due to statins having a greater effect for some people than others. There was evidence of heterogeneity in the CoVs across trials (I^2^ = 67.9%).

## DISCUSSION

We have presented methods for examining differences in outcome variance between the two arms in an RCT, to identify heterogeneity of effects of the intervention. We have added to existing methods by: showing how to use regression-based methods to examine the effects of covariates on variation, where individual participant data are available; applying a difference of variances test to summary data in meta-analyses, alongside the ratio of variances, log of the ratio of SDs, and log of the ratio of the coefficient of variation methods already existing; and noted when the CoV test is not appropriate. We suggest that CoV comparisons only be made where the outcome has a meaningful zero and is on a ratio scale.

Differences in variance could be caused by many factors. One is the existence of patient characteristics that influence the effectiveness of the intervention (effect modifiers), which could manifest as subgroups between which the intervention (or control) treatments have different effects.^9^ For example, the intervention may have a different effect in those with worse (or better) values at baseline, or outcomes in the control arm may vary due to differences in “usual practice.” If there are differences in variance, further studies may be needed to find the effect modifiers that define the subgroups.

Other potential explanations for differences in variance between arms of a trial are noncompliance with the intervention; subgroups that are differently engaged with the intervention (e.g., therapist effects) or an intervention that impacts on within-person variability.^9^ Investigation of other factors relating to variation would require individual or stratified summary data on these factors, such as pretreatment severity, or marital status moderating the response to CBT.^36^ Another explanation for differences in variance is model misspecification (e.g., if the errors follow a nonnormal distribution, or if the errors are not independently distributed). Investigation of misspecification of the model would require individual patient data for each trial.

Simulations confirmed that power to detect heterogeneity in single trials was low unless the trial was very large.^2^ RCTs would need to increase their sample size dramatically to be powered to detect differences in variance. This might be prohibitively expensive in time and money, and it may not be feasible to recruit enough individuals to the trial.^37^ In this case, powering the trial on the main effect is appropriate, and improved reporting, giving detailed summary data across both trial arms, would allow meta-analysis of differences in variance.

We observed smaller variance in the intervention than the control arm in both meta-analyses presented here, but without individual participant data, it was not possible to explore this further. With individual data, the factors associated with the variance can be examined directly, as in our single trial example.^30^ These factors might be used to predict the effect of the intervention in external populations or applied in personalized medicine. The slightly lower variance in the intervention arm in the single trial^30^ and meta-analysis of effects of CBT in depression^33^ may also be partly because the outcome scale (BDI) is bounded at 0 and floor (or ceiling) effects can reduce variance.

A possible cause of differences in variance between two arms of a trial is that the variance is related to the mean, and the intervention causes a mean difference in the outcome. This is clearly shown in our second meta-analysis example, examining the effect of statins on LDL cholesterol.^34^ There was evidence that the variance of the outcome was lower in the intervention than the control arm, implying that statins had a greater effect on those with initially higher cholesterol levels. The CoV results indicated that the variance in the intervention arm was actually a little larger than would have been expected, given the difference in means. This provided (weak) evidence that there was heterogeneity in the effect of statins on LDL cholesterol, but that this was not due to statins having a bigger effect on those with higher cholesterol levels.

It is important to use the right method for the data. If individual participant data were available, Levene and Glejser tests could be used, and comparing results across tests would explore the impact of any non-normality of the data. For meta-analysis of individual trials, the assumption of normality should be checked as far as possible (e.g., by using data presented within each paper such as mean, median, and SD). Expert knowledge could be used to identify outcomes that may be less likely to be normally distributed, for example ratio scale data which are bounded at zero, or outcomes such as body mass index that tends to be skewed. Ratios of variance are appropriate where different scales are used across different trials or where the same scale is used but the mean is very different, as in these situations a difference in variances test may not be appropriate. These methods may be biased when the arms are not independent (e.g., in crossover trials).^12^ The approaches we have used are consistent and asymptotically unbiased, and there are corrections available for small sample sizes.^12^ Where there are three or more repeated measures within a trial (e.g., baseline and posttreatment) then multilevel models (also known as random effects or linear mixed models) could be used. These are robust to data missing at random (i.e., if missingness depends on observed variables) and could explore variation in the rate of change between the arms of the trial.

These methods for quantifying variance between treatment arms are applicable not just to RCTs, but also to differences in variance of continuous outcomes according to genotype in genetic epidemiologic studies.^38–40^ Differences by genotype can be considered as analogous to differences by treatment arm in an RCT,^41,42^ indeed the progenitor of RCTs, RA Fisher, considered the factorial nature of Mendelian inheritance to be the model for randomization in experiments.^43–45^ Difference in variance by allele count at, for example, a single-nucleotide polymorphism locus, is taken as evidence of the presence of either epistasis or gene–environment interaction.^38–40^ A second potential application is within Mendelian randomization implemented within an instrumental variables analysis framework.^46,47^ An interpretative issue relates to the assumption of homogeneity of the effect of the instrument on the exposure, since violations of this would suggest that the effect estimates may not apply to the entire study sample. As nonhomogeneity in the genetic variant—exposure association would lead to nonhomogeneity in the genetic variant—outcome association, then as long as either the exposure or outcome allow variance estimation, an umbrella test of presence and degree of violation of the assumption of homogeneity is possible. This approach would, of course, apply to instrumental variable analysis in general and not just when this is within a Mendelian randomization context.

While conclusions from randomized trials are usually expressed in terms of average effects of an intervention, individuals will want to know how well they personally will respond to an intervention. Grouping subjects according to an observed response is open to bias.^48^ An alternative way to examine variation in response, without having to specify and measure effect modifiers, is to examine differences in variability between the trial arms. We have described different ways of doing this with individual participant or summary data. Given the low power to explore heterogeneity of variance in individual trials, we suggest that meta-analyses should be used where possible. It is important to consider scale when deciding whether to meta-analyze differences or ratios of variance: if all trials use the same outcome scale then it may be plausible to assume that the trials come from a population with a constant difference in variances. If different scales are used, then this is unlikely—but in this case, the ratio of variances could be meta-analyzed. Where appropriate (i.e., the outcome measure is a ratio scale with a true zero) then it is important to examine the coefficient of variation. If evidence of a difference in variation between arms of the trials is found, then effect heterogeneity is not the only explanation—it is important to consider the other explanations such as differences in compliance or model misspecification: using multiple different approaches with individual participant data can help explore these possibilities.

## Acknowledgments

We thank Luke Prendergast for providing example code based on his 2016 paper “Meta-analysis of ratios of sample variances.”

## Supplementary Material


