# Supplementary material for: Detecting Heterogeneity of Intervention Effects Using Analysis and Meta-analysis of Differences in Variance Between Trial Arms
Source: Epidemiology. 2021 Aug 20;32(6):846–54. doi: 10.1097/EDE.0000000000001401 (PMC8478324; doi:10.1097/EDE.0000000000001401)
Supplement: Supplementary file 1 [file ede-32-846-s001.pdf]

Supplemental Digital Content: Detecting heterogeneity of intervention effects using analysis and meta-analysis of differences in variance between trial arms

Authors: Harriet L Mills[a,b]*, Julian PT Higgins[a,b,c], Richard W Morris[b], David Kessler[b,c], Jon Heron[a,b], Nicola Wiles[b,c], George Davey Smith[a,b], Kate Tilling[a,b,c]

Affiliations:

[a]Medical Research Council Integrative Epidemiology Unit, Bristol Medical School, University of Bristol, Bristol, UK

[b]Population Health Sciences, Bristol Medical School, University of Bristol, Bristol, UK

[c]National Institute for Health Research Bristol Biomedical Research Centre, University Hospitals Bristol NHS Foundation Trust and University of Bristol.

Table of Contents

1. Table of Studies 3

2. Methods for examining difference in variance between trial arms – extension to main text methods 6

  2.1 Examining differences in variance between two arms using data from one trial 6

  2.2 Examining the relationship between mean and variation across the two arms 9

3. CoV Simulation Study 11

  3.1 Methods 11

  2.2 Results 11

4. Analysis of a single trial 14

  4.1 Methods 14

  4.2 Results 14

5. Meta-Analyses 16

  5.1 Results 16

6. Power Simulation Study 21

  6.1 Methods 21

  6.2 Results 22

References 24

1. Table of Studies

eTable 1: Summary of the findings of studies using meta-analysis to examine variation between arms, as cited in the introduction of the main text.

Trial	Statistic	Topic	Outcome measure	Number of studies in meta-analysis	Finding
Cally [1]	logRoCV	Sexual selection and population fitness	"fitness components measured in females under stressful conditions"	27	*"under stressful conditions, sexual selection tends to reduce the phenotypic variance in fitness traits"* logRoCV = -0.78 (95%CI -1.23, -0.34) for females; for mixed sex it is similar logRoCV=-0.76 (-1.22, -0.31)
Chamberlain [2]	logRoSD (=logVR)	Visuospatial ability in people with dyslexia	Performance in high-level visuospatial tasks	97 effect sizes	Dyslexia is associated with a greater variability in performance on visuospatial tasks logRoSD = 0.102 (SE=0.0366, p=0.0108)
Munkholm [3]	logVR logRoCV	Individual response to antidepressants for depression in adults	Hamilton Depression Rating Scale or the Montgomery-Åsberg Depression Rating Scale	345 comparisons from 222 RCTs	No evidence for a larger variance in the antidepressant arm compared with placebo overall
O'Dea [4]	logRoCV	Gender differences in academic grades at school	Academic grades	346 effects sizes extracted from 227 studies	There is less variation in girls' grades in STEM subjects than boys', at school: logRoCV = -0.114 (-0.133, -0.095)

Pillinger [5]	logVR, logRoCV	Immune parameters in psychosis	Levels of peripheral immune parameters (eg. Level of blood cytokines)	35	For two immune parameters there is lower variance in control arm.<br>For one immune parameter there is lower variance in intervention arm.
Plöderl [6]	logVR logRoCV	Personalised treatment with anti-depressants	Hamilton Depression Rating Scale or the Montgomery-Åsberg Depression Rating Scale	163 randomised, placebo-controlled trials	No evidence for larger variance in the arms receiving antidepressants compared with the control arm, for any antidepressant.
Prendergast [7]	F-test	Is there a difference in mean spinal bone mass density across genotype groups in pre-menopausal women (illustration of their method)	Mean spinal bone mass density	13	MLE 1.36 (1.03)<br>REML 1.34 (1.00, 1.79)
Senior [8]	logVR, logRoCV	Dietary restriction and longevity	Mean longevity	"77 effect sizes of mean longevity from 21 studies across 14 species" from English and Uller [9]	positive, but not "statistically significant", increase in variance in the arm with dietary restrictions<br>logVR = 0.05 (95% CI -0.045, 0.154)<br>logRoCV=0.09 (-0.021, 0.205)

Senior [10]	logVR, logRoCV, logSD	Effect of two dietary interventions on variability in weight (illustration of methods)	Body mass (kg)	16	Not "statistically significant" - but low carbohydrate diets result in more variance in weight than calorie restricted diet:<br>logVR = -0.08 (-0.19, 0.02)<br>logRoCV = -0.10 (-0.20, 0.9x10^-3)
Williamson [11]	*"true individual response variance"*	Weight change in response to an exercise intervention	Weight change (kg)	12	There is greater variability in weight change in the exercise arm, but it is not "significant":<br>SD_IR = 0.8 (-0.9, 1.4) kg
Winkelbeiner [12]	logVR	RCTs of anti-psychotic drugs in patients with schizophrenia	Syndrome scale	52	Lower variation in intervention arm<br>logVR = 0.97 (95%CI 0.95, 0.99)

2. Methods for examining difference in variance between trial arms – extension to main text methods

In the following two sections we describe in full the methods summarised in Table 1 of the main text.

Throughout the paper we use the following notation. We assume each RCT has two groups, referred to as control (i=0) and intervention (i=1). The groups are of size N_0 and N_1, respectively, where N=N_0+N_1 is the total sample size of the trial. The jth individual in the trial has group allocation Z_j (=0 or 1), and a response Y_j. Let μ_i and σ_i^2 be the underlying mean and variance of responses Y_j for individuals in group i, with sample estimates denoted by μˆ_i and σˆ_i^2.

2.1 Examining differences in variance between two arms using data from one trial

Glejser's test. The test proposed by Glejser [13] takes the absolute value of the residuals (ϵ_i) from the standard linear model:

$$Y_j = \beta_0 + \beta_1 Z_j + \epsilon_j,$$

and regresses them on the explanatory variable (in this instance, the arm indicator Z_j):

$$|\epsilon_j| = \gamma_0 + \gamma_1 Z_j + v_j$$

A one-sample t-test based on $\hat{\gamma}_1$ of whether $\gamma_1 = 0$ is used to test the null hypothesis that the variances in the two arms are the same. The linear model can include covariates, and thus examine whether known covariates explain the differences in variance.

Levene's test. Levene's test is suitable for non-normally distributed data (and may be less powerful than the alternatives for normally distributed data) and can be based on absolute deviations from the median, mean or trimmed mean [14]. For the two trial arms, using the notation defined above, the test statistic is calculated as:

$$W = (N-2) * \frac{\sum_{i=0}^{1} N_i (X_{i.} - X_{..})^2}{\sum_{i=0}^{1}\sum_{j=1}^{N_i} (X_{ij} - X_{i.})^2}$$

where, within each arm ($i = 0{,}1$), we define $X_{ij} = |Y_j - m_i|$ (i.e. the absolute deviations where m_i is either the mean (μ_i), the median (resulting in the Brown-Forsythe test [15]) or the trimmed mean of responses in the ith arm), $X_{i.} = \frac{1}{N_i}\sum_{j=1}^{N_i} X_{ij}$ is the mean of the X_{ij} within arm i and $X_{..} = \frac{1}{N}\sum_{i=0}^{1}\sum_{j=1}^{N_i} X_{ij}$ is the mean of all X_{ij}.

The test statistic has an approximate F-distribution with 1 and $N-2$ degrees of freedom. It is noted that Levene's test can also be performed using a regression framework (as with Glejser's test, but using least absolute deviation regression [16]), in which case an estimate of the difference in variation can be obtained alongside a p-value. As with Glesjer's test, the initial least absolute deviation regression model can be extended to include other covariates.

Bartlett's test. The equality of variances between two arms can be tested using Bartlett's test [17]. This involves a test statistic, T_B, calculated as:

$$T_B = \frac{(N-2)\ln\left(\hat{\sigma}_p^2\right) - ((N_0-1)\ln(\hat{\sigma}_0^2) + (N_1-1)\ln(\hat{\sigma}_1^2))}{1 + \frac{1}{3}\left(\left(\frac{1}{(N_0-1)} + \frac{1}{(N_1-1)}\right) - \frac{1}{N-2}\right)}$$

where $\hat{\sigma}_p^2 = \frac{1}{N-2}\sum_{i=0}^{1}(N_i-1)\hat{\sigma}_i^2$ (the weighted estimate for the variance).

The test statistic, T_B, has an approximate χ_1^2 distribution when the variances are equal. Bartlett's test assumes that the underlying distributions in each arm of the trial are Normal.

Estimating parameters from a linear model with non-constant variance (LMNCV). The standard linear model for a two-arm trial with a continuous outcome assumes that the variances are equal in the two arms, such that $\sigma_0^2 = \sigma_1^2 = \sigma^2$:

$$Y_j = \beta_0 + \beta_1 Z_j + \epsilon_j,$$

with

$$\epsilon_j \sim N(0, \sigma^2).$$

Here β_0 $(= \mu_0)$ is the mean in the control arm and β_1 is the difference in means between the arms $(\mu_1 - \mu_0)$. Omitting the intercept, we can write this using the notation above as

$$Y_j = \mu_0\left(1 - Z_j\right) + \mu_1 Z_j + \epsilon_j,$$

$$\epsilon_j \sim N(0, \sigma^2).$$

We can extend this formulation to allow the variances to differ between the two arms:

$$Y_j = \mu_0\left(1 - Z_j\right) + \mu_1 Z_j + \epsilon_{0j} Z_j\left(1 - Z_j\right) + \epsilon_{1j} Z_j,$$

with

$$\epsilon_{ij} \sim \mathrm{N}\left(0, \sigma_i^2\right), \text{ for } i = 0, 1.$$

This model can be re-expressed in the form of a linear mixed model (LME) as follows, facilitating implementation in mixed modelling software:

$$Y_j = \beta_0 + \beta_1 Z_j + u_j Z_j + \epsilon_j,$$

with

$$\epsilon_j \sim \mathrm{N}(0, \sigma_\epsilon^2) \; \& \; u_j \sim \mathrm{N}(0, \sigma_u^2).$$

Here, σ_ϵ^2 $(= \sigma_0^2)$ is the variance in the control arm and σ_u^2 is the difference in variance between the arms $(\sigma_1^2 - \sigma_0^2)$. To estimate the parameters from this form of the model freely, software must allow variances to be negative. Since many software packages require all variances to be positive, the mixed model parameterisation would require that the model be specified with the arm with larger variance as arm 1. Whichever formulation of the model is used, post-estimation confidence intervals (or credible intervals if a Bayesian framework is

used) can be derived for either the difference or the ratio of the two variances. Both formulations also can include covariates and thus can be used to investigate the known factors that might explain the difference in variances.

Estimating the magnitude of difference (DiV). The magnitude of the difference between the two variances can be estimated by taking the difference of the sample variances. The difference in variances (DiV) is obtained by simple subtraction:

$$\text{DiV} = \sigma_1^2 - \sigma_0^2$$

The approximate standard error (SE) of each estimated variance, $\hat{\sigma}_i^2$, is [18]:

$$\text{SE}_{\sigma_i^2} = \hat{\sigma}_i^2 \sqrt{\frac{2}{N_i - 1}}$$

Since in a two-arm trial the two arms are independent, the SE of the DiV is given by

$$\text{SE}_{\text{DiV}} = \sqrt{SE_{\sigma_0^2}^2 + SE_{\sigma_1^2}^2} = \sqrt{2\left(\frac{\hat{\sigma}_0^4}{N_0 - 1} + \frac{\hat{\sigma}_1^4}{N_1 - 1}\right)}.$$

The variability of the two arms is compared by a t-test, with test statistic $\text{DiV}/\text{SE}_{\text{DiV}}$, where the SE_{DiV} is calculated under the null hypothesis, i.e. assuming that $\hat{\sigma}_1^2 = \hat{\sigma}_0^2 = \frac{(N_0-1)s_0^2 + (N_1-1)s_1^2}{(N_0+N_1-2)}$

This method relies on the samples being sufficiently large that the chi-square distribution for the variance can be approximated by a Normal distribution. The distribution of the variance is chi-squared, and thus asymmetric - but for larger samples (>=100) can be approximated by a Normal distribution.

The ratio of variances method (F-test, RoV). A simple F statistic formed by the ratio of sample variances between the two arms,

$$F = \frac{\hat{\sigma}_0^2}{\hat{\sigma}_1^2}$$

follows the F-distribution with $N_0 - 1$ and $N_1 - 1$ degrees of freedom if the true variances of two normally distributed variables are equal, so can be used to test for equality of the two variances (assuming that the outcome is Normally distributed in both groups). The F-distribution can be used to derive a confidence interval for the RoV.

Log of the ratio of standard deviations (logRoSD). The log of the ratio of standard deviations can be used to compare variance between two arms [12,19]; logRoSD is calculated as the log of the ratio of standard deviations:

$$logRoSD = log\left(\frac{\sigma_1}{\sigma_0}\right) + \frac{1}{2(N_1 - 1)} - \frac{1}{2(N_0 - 1)}$$

with sampling variance

$$\sigma^2_{logRoSD} = \frac{1}{2(N_1 - 1)} + \frac{1}{2(N_0 - 1)}$$

(Note that this is called the log of the variability ratio, and referred to as logVR in [12,19] but to avoid notation confusion we have used RoSD to reflect that it is the ratio of standard deviations.)

The variability of the two arms is compared by a t-test on logRoSD (i.e., the test statistic is $\mathrm{logRoSD}/\sigma_{\mathrm{logRoSD}}$).

2.2 Examining the relationship between mean and variation across the two arms

Difference in coefficient of variation (DiCV). For arm i with mean μ_i and SD σ_i the CoV is estimated as:

$$\mathrm{CoV}_i = \frac{\hat{\sigma}_i}{\hat{\mu}_i}$$

We use the method described by Feltz and Miller [20] to compare the CoV of two arms. A pooled CoV across the arms is

$$\mathrm{CoV}_p = \frac{(N_0 - 1)\mathrm{CoV}_0 + (N_1 - 1)\mathrm{CoV}_1}{N_0 + N_1 - 2},$$

and the test statistic is

$$Z = \frac{\mathrm{CoV}_0 - \mathrm{CoV}_1}{\sqrt{\left(\frac{\mathrm{CoV}_p^2}{N_0 - 1} + \frac{\mathrm{CoV}_p^2}{N_1 - 1}\right)(0.5 + \mathrm{CoV}_p^2)}}.$$

Z^2 approximates the chi-square distribution with one degree of freedom. This method performs best if each $N_i > 10$ and each $\mathrm{CoV}_i > 0.33$ [20].

The standard error of the difference in the coefficient of variation (not under the null hypothesis), $\mathrm{SE}_{\mathrm{DiCV}}$ is given by: $\sqrt{\left(\frac{\mathrm{CoV}_0^2}{N_0 - 1}\right)(0.5 + \mathrm{CoV}_0^2) + \left(\frac{\mathrm{CoV}_1^2}{N_1 - 1}\right)(0.5 + \mathrm{CoV}_1^2)}$.

Log of the coefficient of variation ratio (logRoCV). Using the CoV as calculated above, the log of the ratio of coefficient of variations can be calculated and used to compare differences in variability between the two arms [19]:

$$logRoCV = log\left(\frac{CoV_1}{CoV_0}\right) + \frac{1}{2(N_1 - 1)} - \frac{1}{2(N_0 - 1)}$$

where $CoV_i = \sigma_i/\mu_i$. As logRoCV uses the CoV, it should only be used when data satisfies the same criteria as for using CoV (data on a ratio scale, with a meaningful zero).

The sampling variance is defined

$$\sigma_{logRoCV}^2 = \frac{\sigma_0}{N_0\mu_0^2} + \frac{1}{2(N_0 - 1)} - 2\rho_{log\mu_0,log\sigma_0}\sqrt{\frac{\sigma_0^2}{N_0\mu_0^2}\frac{1}{2(N_0 - 1)}}$$

$$+ \frac{\sigma_1}{N_1\mu_1^2} + \frac{1}{2(N_1 - 1)} - 2\rho_{log\mu_1,log\sigma_1}\sqrt{\frac{\sigma_1^2}{N_1\mu_1^2}\frac{1}{2(N_1 - 1)}}$$

where $\rho_{log\mu_i,log\sigma_i}$ are the correlations between the means and standard deviations (on log scales) across studies, for the control ($i = 0$) and intervention ($i = 1$) arms.

These rho terms can be removed if we make the assumption that the data are normally distributed (as in the R package that implements these equations for meta-analysis, *metafor*). In this work, we assume normality and therefore remove the rho terms.

The variances of the two arms are compared by a t-test on logRoCV (i.e., the test statistic is $logRoCV/\sigma_{logRoCV}$).

3. CoV Simulation Study

3.1 Methods

Data are simulated for 20 trials with 100 observations in each, as follows:

Scenario 1 (same CoV in each arm), for each trial:

1. Randomly assign 200 observations to treatment, T=0 or T=1, with probability 0.5
2. Generate the "true" effect of treatment for trial j, as $\alpha_j = \mu_j + 10$ where μ_j is drawn from a normal distribution $N(0,1)$ (i.e $\alpha_j \sim N(10,1)$)
3. Generate observed outcomes for individual i in trial j, so that the CoV is 0.5 in each arm:

$$if\ T = 0: Outcome_{ij} = \alpha_j + \alpha_j \times 0.5 \times \beta_i$$

$$if\ T = 1: Outcome_{ij} = (\alpha_j + 10) + (\alpha_j + 10) \times 0.5 \times \beta_i$$

$$where\ \beta_i \sim N(0,1)$$

4. Record the N in each arm and calculate the mean and SD of each arm.
5. Repeat over 20 trials

Scenario 2 (different CoV in each arm), for each trial:

1. Randomly assign observations to treatment, T=0 or T=1, with probability 0.5
2. Generate the "true" effect of treatment for trial j, as $\alpha_j = \mu_j + 10$ where μ_j is drawn from a normal distribution $N(0,1)$ (i.e $\alpha_j \sim N(10,1)$)
3. Generate observed outcomes for individual i in trial j, so that the CoV is 0.5 in each arm:

$$if\ T = 0: Outcome_{ij} = \alpha_j + \alpha_j \times 0.5 \times \beta_i$$

$$if\ T = 1: Outcome_{ij} = (\alpha_j + 10) + (\alpha_j + 10) \times 1 \times \beta_i$$

$$where\ \beta_i \sim N(0,1)$$

6. Record the N in each arm and calculate the mean and SD of each arm.
7. Repeat over 20 trials

Each scenario was then analysed as follows:

Across the 20 trials, (1) correlations between mean and SD of each arm were calculated; (2) mean and SD for each arm were plotted against one another; (3) coefficient of variation was calculated for both arms of each trial; (4) these CoVs were meta-analysed, as described in the main paper.

Code (in R) is included with this paper for these simulations.

2.2 Results

The seed is fixed at the start of these scenarios, because of this, the scenarios have the same correlation between mean and SD in the control arms (T=0), eTable 2.

eTable 2: Correlation between mean and SD and mean CoV for the simulated trials in each scenario.

	**Scenario 1**		**Scenario 2**	
	**T=0**	**T=1**	**T=0**	**T=1**

Correlation between mean and SD	0.53	0.38	0.53	0.27
Mean CoV	0.48	0.48	0.48	0.95
Mean of the outcome means	10.12	20.70	10.12	21.43
Mean of the outcome SDs	4.85	9.92	4.85	19.84

eFigure 1 and eFigure 2 plot the mean against the SD from the two arms, for each scenario, and include unadjusted regression lines (with intercept and forced through the origin). The unadjusted regression line is not helpful for interpreting the coefficient of variation. For example, it may seem that the mean and SD are not related when in fact they are, because of regression dilution bias. This could be mitigated by using the regression line forced through the origin (shown in orange below).

eFigure 1: Plot of mean outcome vs SD for scenario 1. The correlation coefficient is given as r, in the bottom right of each plot. Blue solid line: the unadjusted regression line and 95% confidence intervals. Orange dashed line: the unadjusted regression line, forced through the origin, with 95% confidence intervals.

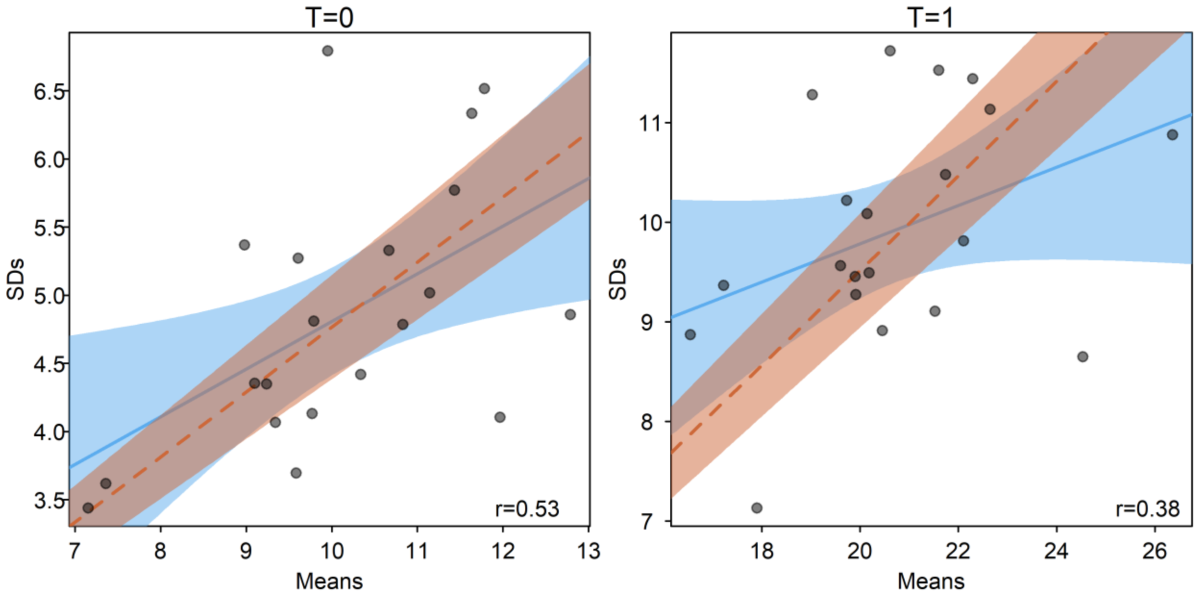

eFigure 2: Plot of mean outcome vs SD for scenario 2. The correlation coefficient is given as r, in the bottom right of each plot. Blue solid line: the unadjusted regression line and 95% confidence intervals. Orange dashed line: the unadjusted regression line, forced through the origin, with 95% confidence intervals.

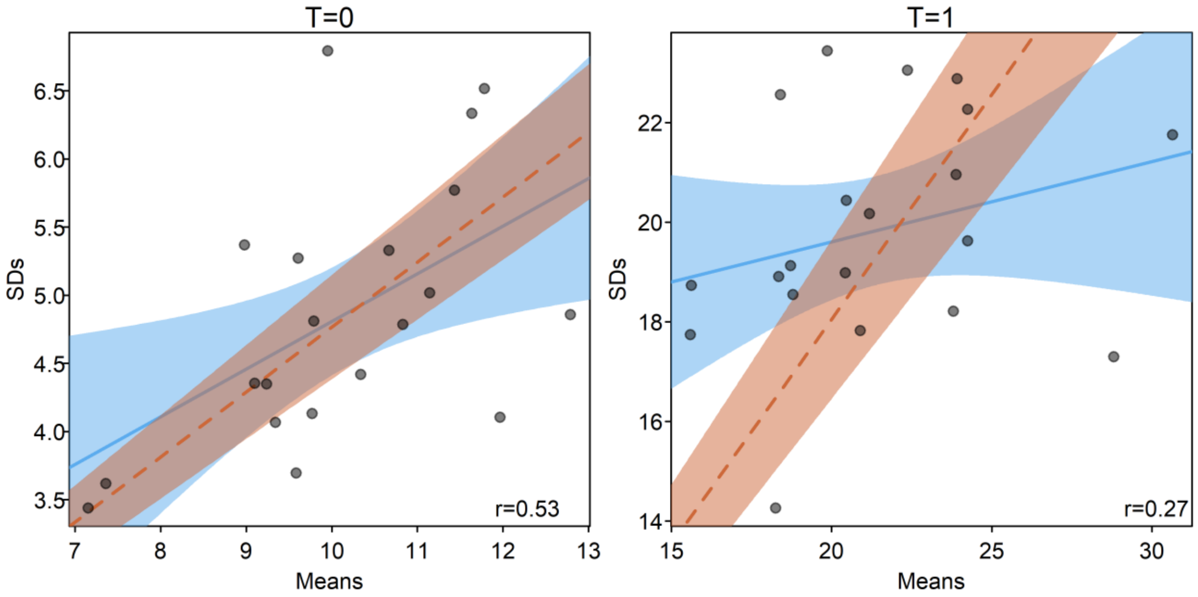

The meta-analysis shows that the coefficient of variation behaves quite differently in the two scenarios. In scenario 1, the CoVs are around 0.5 in both arms, and the meta-analysis estimates imply that differences in variation between arms across the trials may be due to differences in the means. In scenario 2, the CoV are different between the arms and the meta-analysis estimates imply that differences in variation between arms were not just due to differences in the means, eTable 3.

eTable 3: Meta-analysis of the Difference in CoV for the simulated trials from each scenario.

Meta analysis		Estimate	Standard Error	Lower	Upper	Pvalue
Scenario 1	**Fixed**	-0.0063	0.0182	-0.0421	0.0294	0.7285
	**Random**	-0.0052	0.0216	-0.0474	0.0371	0.8100
Scenario 2	**Fixed**	-0.4246	0.0309	-0.4853	-0.3640	0.0000
	**Random**	-0.4462	0.0428	-0.5300	-0.3624	0.0000

These scenarios demonstrate how the correlation plots tell us nothing about the CoV across the meta-analysis. The decision to compare CoV between the two arms (or to meta-analyse the difference in CoV across trials) should be based on substantive grounds (i.e. are there grounds to believe that the variance changes with the mean). If there is no evidence of a difference in variance between the two arms then a comparison of the CoV may not be meaningful. For example, if the treatment effect is homogeneous, then the variation in the two arms would be the same but the means would differ, leading to a difference in CoV. However, this difference would not be of clinical relevance.

4. Analysis of a single trial

4.1 Methods

eFigure 3: Beck Depression Inventory (BDI) score at baseline (A) and at 4-month follow-up (B) in Kessler et al [21]. The 4-month BDI scores are not normally distributed. Colours indicate the different arms (darker red where they overlap).

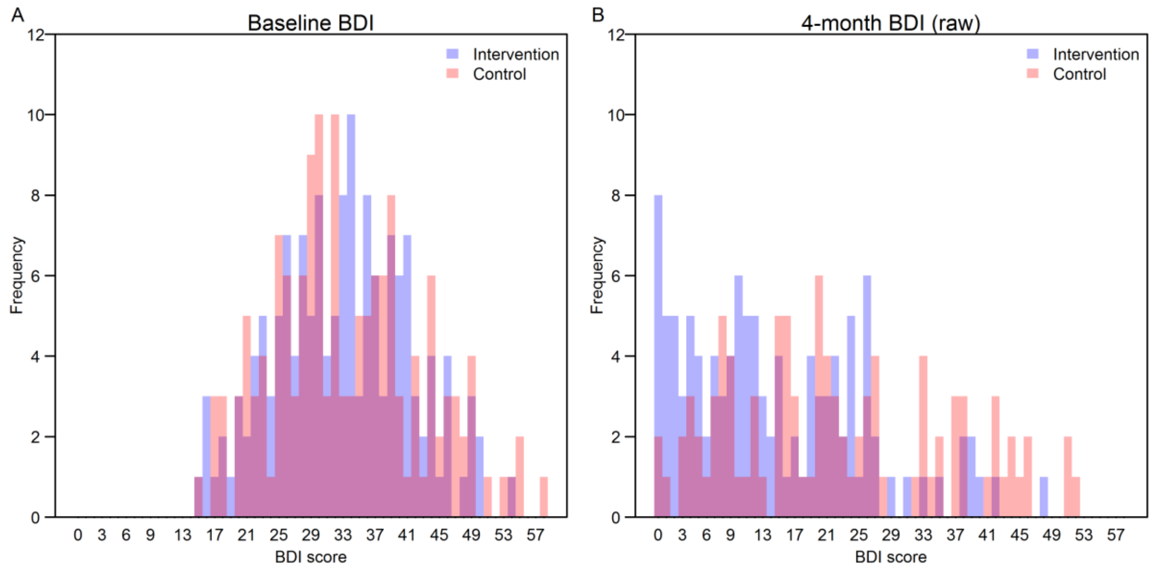

4.2 Results

Koenker's test

We note that Koenker's test (the studentized version of the Breusch-Pagan test) [22] is similar to Glejser's test as described above, but takes the square of the residuals instead of the absolute value. Glejser's test does not provide direct estimates of the difference in variance (or the SE and CI) but Koenker's test can. As a supplement to Table 3 in the main text, we note that Koenker's test supports our main conclusions that including baseline BDI score (adjusted model 2, in eTable 4) largely removed any evidence of difference in variance between the arms.

eTable 4: Tests for difference in variance in BDI score at 4 months, between the intervention and control arms from the single trial exploring the effect of a CBT intervention on depression [21]

Test	Test Statistic		p-value	Estimate	95% CI
Koenker's test, unadjusted	t-statistic	2.17	0.031	-55.98	(-106.75, -5.23)
Koenker's test, adjusted 1[a]	t-statistic	2.42	0.016	-61.23	(-111.05, -11.40)
Koenker's test, adjusted 2[b]	t-statistic	1.13	0.26	-19.38	(-53.07, 14.31)

[a] Covariates added in the adjusted model 1 are as specified in the original trial paper: centre ID, present antidepressant treatment, sex, whether or not GP practice has a counsellor
[b] As adjusted model 1, but also including baseline BDI score

Sensitivity Analysis

eTable 5 shows the results of all tests on the IPD at baseline. The results for all tests are similar, with no evidence for any difference in variance between the intervention and control arm, even when testing only the subset remaining after excluding those lost to attrition at 4 month follow up. The intervention arm had 24% attrition at 4 months, compared to 34% in the control arm.

eTable 5: Tests for difference in variance in BDI score at baseline, between the intervention and control arms from the Kessler 2009 paper [21] exploring the effect of a CBT intervention on depression. The test statistics are the Bartlett's k-squared for Bartlett's test, the ratio of variances for the F-test and the Levene test-statistic for Levene's test.

Test	Test Statistic	p-value	Estimate	SE
Baseline				
Levene test (median)	1.468	0.23	0.735	0.607
Levene test (mean)	1.904	0.17	0.808	0.585
Levene test (trimmed mean)	1.659	0.20	0.762	0.592
Bartlett's test	1.655	0.20	*NA*	*NA*
F-test	0.809	0.20	*NA*	*NA*
Baseline, excluding those lost to attrition by 4 months				
Levene test (median)	2.453	0.12	1.083	0.691
Levene test (mean)	2.618	0.11	1.111	0.686
Levene test (trimmed mean)	2.515	0.11	1.092	0.688
Bartlett's test	1.637	0.20	*NA*	*NA*
F-test	0.777	0.20	*NA*	*NA*

5. Meta-Analyses

5.1 Results

eTable 6: Results for the Richards et al [23] meta-analyses (self-reported depression measures). Yellow shading in each row indicates the arm with the higher SD. Estimates for the Ratio of Variances and Log of Variability ratio are also plotted in Figure 1. The final rows show the results of the pooled RoV test, and the meta-analysis of the Differences in Variance tests.*

Study	Measure used	Intervention arm			Control arm			Difference in Variances test [95% CI]	Ratio of Variances [95% CI]	Log of variability ratio [95% CI]
		Mean	SD	N	Mean	SD	N			
Andersson et al 2005	BDI	12.2	6.8	36	19.5	8.1	49	-19.37 [-53.40, 14.66]	0.70 [0.38, 1.34]	-0.17 [-0.48, 0.14]
De Graaf et al 2009&2011	BDI	20.6	10.4	97	22.1	10.2	97	4.12 [-38.34, 46.58]	1.04 [0.70, 1.55]	0.02 [-0.18, 0.22]
Hollandare et al 2011	BDI	9.3	12	38	13.4	11.9	39	2.39 [-89.04, 93.82]	1.02 [0.53, 1.95]	0.01 [-0.31, 0.33]
Kessler et al 2009	BDI	14.5	11.2	113	22	13.5	97	-56.81 [-117.95, 4.33]	0.69 [0.47, 1.01]	-0.19 [-0.38, 0.01]
Meyer et al 2009	BDI	19.87	11.85	159	27.15	10.01	57	40.22 [-8.11, 88.56]	1.40 [0.89, 2.12]	0.16 [-0.05, 0.38]
Perini et al 2009	BDI	17.3	9.86	27	23.33	9.29	17	10.92 [-68.89, 90.72]	1.13 [0.43, 2.66]	0.05 [-0.39, 0.49]
Proudfoot 2003&2004	BDI	12.1	9.3	95	18.4	10.9	100	-32.32 [-73.63, 8.99]	0.73 [0.49, 1.09]	-0.16 [-0.36, 0.04]
Ruwaard et al 2009	BDI-IA	9.8	6.5	36	15.6	7.6	18	-15.51 [-59.09, 28.07]	0.73 [0.30, 1.60]	-0.17 [-0.58, 0.24]
Spek et al 2007&2008	BDI	11.97	8.05	102	14.46	10.42	100	-43.77 [-78.91, -8.64]	0.60 [0.40, 0.88]	-0.26 [-0.45, -0.06]
Titov et al 2010	BDI-II	15.29	9.81	41	26.15	10.14	40	-6.58 [-68.72, 55.56]	0.94 [0.50, 1.76]	-0.03 [-0.35, 0.28]
Vernmark et al 2010	BDI	10.3	5.2	29	16.6	7.9	29	-35.37 [-71.00, 0.26]	0.43 [0.20, 0.92]	-0.42 [-0.79, -0.05]
All trials										
Fixed								-19.13 [-32.79, -5.48]		
Random								-18.19 [-33.80, -2.58]	0.82 [0.67, 1.00]	-0.10 [-0.20, -0.00]

eTable 7: Results for the Palmer et al [24] meta-analyses, measuring the impact of statins on LDL cholesterol (reported in mg/dL). Yellow shading in each row indicates the arm with the higher SD. Estimates are also plotted in Figure 2. The final rows show the results of the pooled RoV test, and the meta-analysis of the Differences in Variances and CoV tests.

Study	Intervention arm				Control arm				Difference in Variances (DiV) test [95% CI]	Ratio of Variances (RoV) [95% CI]	Coefficient of Variation (CoV) test [95% CI]
	Mean (mg/dL)	SD	N	CoV	Mean (mg/dL)	SD	N	CoV			
Tonolo 1997	127	35	10	0.28	189	37	9	0.20	-144.00 [-1899.25, 1611.25]	0.89 [0.21, 3.67]	0.08 [-0.09, 0.25]
Hommel 1992	100	19	12	0.19	182	39	9	0.21	-1160.00 [-2680.78, 360.78]	0.24 [0.06, 0.87]	-0.02 [-0.16, 0.11]
Nielsen 1993	116	22	8	0.19	166	37	10	0.22	-885.00 [-2247.72, 477.72]	0.35 [0.08, 1.71]	-0.03 [-0.18, 0.12]
Aranda 1994	166	37	8	0.22	208	12	8	0.06	1225.00 [-217.14, 2667.14]	9.51 [1.90, 47.49]	0.17 [0.04, 0.29]
LORD Study 2006	95	35	16	0.37	160	45	18	0.28	-800.00 [-2419.21, 819.21]	0.60 [0.22, 1.70]	0.09 [-0.09, 0.27]
Fried 2001	97	27	6	0.28	124	23	11	0.19	200.00 [-815.68, 1215.68]	1.38 [0.33, 9.12]	0.09 [-0.11, 0.30]
Zhang 1995	100	24	10	0.24	127	29	10	0.23	-265.00 [-1206.81, 676.81]	0.68 [0.17, 2.76]	0.01 [-0.15, 0.17]
Imai 1999	128	23	15	0.18	155	44	19	0.28	-1407.00 [-2731.15, -82.85]	0.27 [0.10, 0.79]	-0.10 [-0.23, 0.02]
Lam 1995	116	31	16	0.27	146	33	18	0.23	-128.00 [-1132.48, 876.48]	0.88 [0.32, 2.48]	0.04 [-0.09, 0.17]
Mori 1992	93	22	18	0.24	126	33	15	0.26	-605.00 [-1474.87, 264.87]	0.44 [0.15, 1.22]	-0.03 [-0.16, 0.11]
Makamura 2002	130	24	20	0.18	216	36	20	0.17	-720.00 [-1621.85, 181.85]	0.44 [0.18, 1.12]	0.02 [-0.06, 0.10]
Verma 2005	80	32	44	0.40	133	44	39	0.33	-912.00 [-1884.19, 60.19]	0.53 [0.28, 0.98]	0.07 [-0.06, 0.20]
Yasuda 2004	127	37	39	0.29	168	36	41	0.21	73.00 [-764.57, 910.57]	1.06 [0.56, 2.00]	0.08 [-0.01, 0.16]
Goicoechea 2006	101	25	44	0.25	126	29	19	0.23	-216.00 [-825.66, 393.66]	0.74 [0.31, 1.55]	0.02 [-0.08, 0.11]

Panichi 2005	104	29	28	0.28	131	21	27	0.16	400.00 [-108.65, 908.65]	1.91 [0.87, 4.14]	0.12 [0.03, 0.21]
Bianchi 2003	121	21	28	0.17	206	21	28	0.10	0.00 [-332.69, 332.69]	1.00 [0.46, 2.16]	0.07 [0.02, 0.12]
Lee 2002	102	18	42	0.18	116	28	40	0.24	-460.00 [-835.18, -84.82]	0.41 [0.22, 0.77]	-0.06 [-0.13, 0.00]
ESPLANADE Study 2010	96	33	92	0.34	132	38	94	0.29	-355.00 [-876.90, 166.90]	0.75 [0.50, 1.14]	0.06 [-0.02, 0.13]
Sawara 2006	99	13	22	0.13	125	17	16	0.14	-120.00 [-350.71, 110.71]	0.58 [0.21, 1.48]	-0.00 [-0.07, 0.06]
UK-HARP-I 2005	85	29	121	0.34	114	33	120	0.29	-248.00 [-597.07, 101.07]	0.77 [0.54, 1.11]	0.05 [-0.01, 0.11]
Di Lullo 2005	87	8	80	0.09	161	23	50	0.14	-465.00 [-675.42, -254.58]	0.12 [0.07, 0.20]	-0.05 [-0.08, -0.02]
PREVEND IT 2000	120	35	375	0.29	151	35	379	0.23	0.00 [-247.64, 247.64]	1.00 [0.82, 1.22]	0.06 [0.03, 0.09]
All trials											
Fixed									-220.36 [-318.84, -121.87]	-	0.02 [0.01, 0.03]
Random									-226.33 [-376.77, -75.90]	0.66 [0.48, 0.91]	0.03 [-0.00, 0.06]
Removing trials N<=10											
Fixed									-223.51 [-323.90, -123.12]	-	0.02 [0.00, 0.03]
Random									-233.17 [-388.82, -77.53]	0.62 [0.44, 0.87]	0.03 [-0.01, 0.06]

eTable 7, continued.

Study	Log of Ratio of Standard Deviation (logRoSD) [95% CI]	Log of ratio of coefficient of variation (logRoCV) [95% CI]
Tonolo 1997	-0.06 [-0.74, 0.61]	0.34 [-0.37, 1.04]
Hommel 1992	-0.74 [-1.38, -0.09]	-0.14 [-0.81, 0.53]
Nielsen 1993	-0.50 [-1.20, 0.19]	-0.15 [-0.87, 0.58]
Aranda 1994	1.13 [0.39, 1.87]	1.35 [0.59, 2.11]
LORD Study 2006	-0.25 [-0.74, 0.24]	0.27 [-0.27, 0.81]
Fried 2001	0.21 [-0.55, 0.97]	0.46 [-0.34, 1.25]
Zhang 1995	-0.19 [-0.84, 0.46]	0.05 [-0.64, 0.73]
Imai 1999	-0.64 [-1.13, -0.15]	-0.45 [-0.97, 0.07]
Lam 1995	-0.06 [-0.55, 0.43]	0.17 [-0.35, 0.69]
Mori 1992	-0.41 [-0.91, 0.09]	-0.11 [-0.64, 0.42]
Makamura 2002	-0.41 [-0.86, 0.04]	0.10 [-0.36, 0.56]
Verma 2005	-0.32 [-0.63, -0.01]	0.19 [-0.16, 0.53]
Yasuda 2004	0.03 [-0.29, 0.34]	0.31 [-0.03, 0.64]
Goicoechea 2006	-0.16 [-0.55, 0.22]	0.06 [-0.35, 0.47]
Panichi 2005	0.32 [-0.06, 0.70]	0.55 [0.15, 0.95]
Bianchi 2003	0.00 [-0.38, 0.38]	0.53 [0.15, 0.92]
Lee 2002	-0.44 [-0.75, -0.13]	-0.31 [-0.64, 0.01]
ESPLANADE Study 2010	-0.14 [-0.35, 0.06]	0.18 [-0.05, 0.40]
Sawara 2006	-0.28 [-0.75, 0.19]	-0.04 [-0.52, 0.43]
UK-HARP-I 2005	-0.13 [-0.31, 0.05]	0.16 [-0.03, 0.36]
Di Lullo 2005	-1.06 [-1.31, -0.81]	-0.44 [-0.70, -0.19]
PREVEND IT 2000	0.00 [-0.10, 0.10]	0.23 [0.12, 0.34]
All trials		
Fixed		

Random	-0.21 [-0.37, -0.05]	0.12 [-0.02, 0.26]
Fixed		
Random	-0.24 [-0.41, -0.08]	0.09 [-0.05, 0.24]

6. Power Simulation Study

6.1 Methods

To explore the power of the methods for detecting a difference in variance, under different scenarios, a simulation study was used.

Simulating data

A response $Y = Y_0 + Z * Y_1$ was simulated for two arms $Z \in (0,1)$ of size N_0 and N_1, where Y_0 was the response in the control arm and Y_1 was the treatment effect. Then for $N = N_0 + N_1$ individuals:

$$Y_0 \sim N(\mu_0, \sigma_0)$$
$$Y_1 \sim N(\mu_1, \sigma_1)$$

Without loss of generality, the variables were standardised to the standard deviation in the baseline arm (arm 0, $\sigma_0 = 1$) with means $\mu_0 = 0$ and $\mu_1 = 1$ and with the standard deviation for Y_1 allowed to vary such that $\sigma_1 \in (0.2, 0.3, \ldots., 1.0)$. The number of individuals in each arm was fixed as $N_0 = N_1 = N/2$.

A single simulated dataset consisted of ID (1 to N), the response Y and an arm indicator Z. Two example simulated datasets for $N = 10{,}000$ are shown in eFigure 4.

eFigure 4: Two simulated datasets of 10,000 responses (5000 in each arm, Z=0 and Z=1). Simulated with $m_0 = 0$, $m_1 = 1$, $\sigma_0 = 1$ and (A) $\sigma_1 = 0.1$ and (B) $\sigma_1 = 1.0$. Red shows $Z = 0$ and blue shows $Z = 1$ (where they overlap is the purple/red).

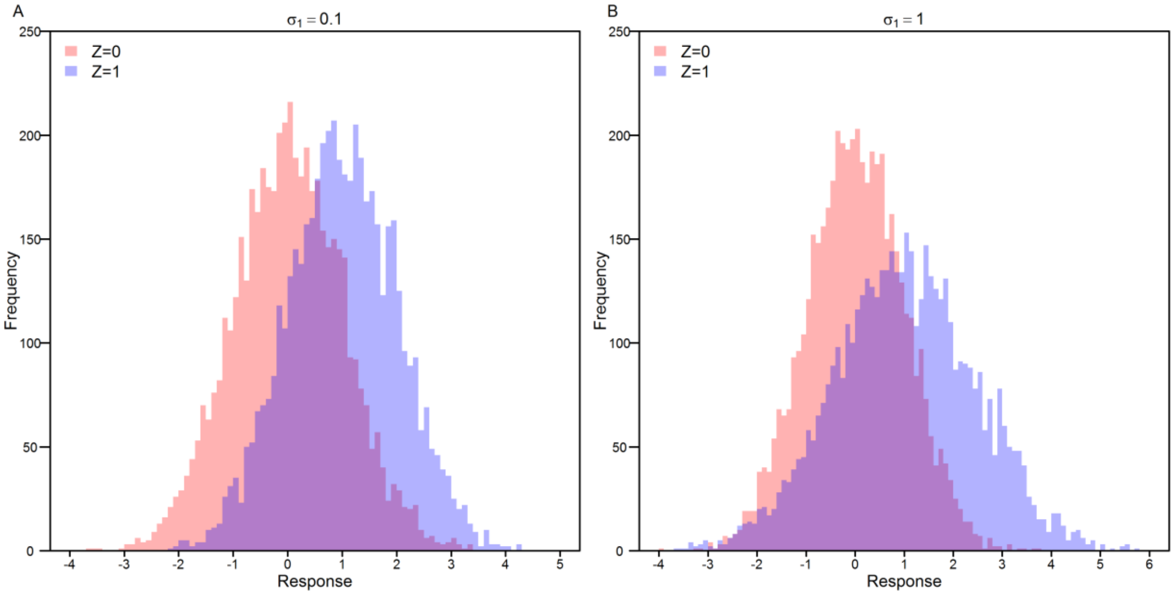

Simulation and analysis process

The aim was to determine what minimum sample size N allowed the difference in variance to be detected with 95% power, for different σ_1 (standard deviation of the treatment effect). σ_1 was varied between 0.2 and 1 (note that this meant the standard deviation of arm Z=1 changed, as it is equal to the square root of the sum of the two standard deviations squared, i.e. $\sqrt{\sigma_0^2 + \sigma_1^2}$). For each σ_1, a binary search algorithm was used first to find what value of N (the total sample size) obtained an approximately 50% power (for efficiency, this uses only 100 simulated datasets). Then, starting at this N, N was increased up to 500,000 (with increasing step sizes) simulating 10,000 datasets for each N. In each of the 10,000 simulated datasets the difference in variance between the two arms was tested using: (1) an LME model; (2) Glejser's test; (3) Levene's test (using deviation from the mean); (4) Bartlett's test; (5) Ratio of Variances (F-test) method. For each N the power was defined as

the percentage of simulations for which the p-value for the test of the null hypothesis (that the difference in variance is zero) was <0.05. N was increased until the power to detect the difference in variance had reached a threshold of 95% for the last three N.

6.2 Results

Power to detect a difference in variance (using an LME model, Glejser's test, Levene's test, Bartlett's test and the Ratio of Variances method) increased with sample size N for all σ_1 scenarios, though much larger sample sizes were required to obtain adequate power when the difference in variance between the arms was low (eFigure 5 & eFigure 6). Results were very similar for all methods, with the Bartlett's test, RoV method and the LMER model requiring very slightly lower sample size for the same power compared to Levene's and Glejser's tests.

eFigure 5: Plot of sample size (N) vs the power to detect difference in variances between the two arms for scenarios with different standard deviations in the two arms (varying σ_1: see methods). The numbers on the lines indicate the value of σ_1. Grey dashed horizontal lines indicate power = 0.8, 0.9, 0.95 and 1.0. 10,000 simulations were performed for each N. (eFigure 6 shows individual panels for each σ_1, without a logged x-axis.)

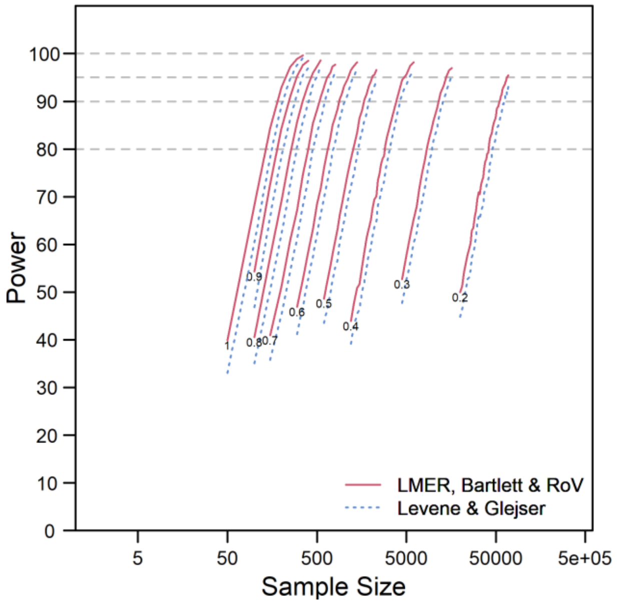

eFigure 6: Plots of N (sample size) vs the power to detect difference in variances between the two arms for scenarios with different standard deviations in the two arms (with fixed $\sigma_0 = 1$ varying σ_1: see methods). Grey dashed horizontal lines indicate power = 0.8, 0.9, 0.95 and 1.0. 10,000 simulations were performed for each N. This is an alternative version of eFigure 5. All methods are plotted, but the results are the same for (1) LMER, Bartlett's test and RoV (the purple/red, top line) and (2) Levene and Glejser tests (the blue, bottom line).

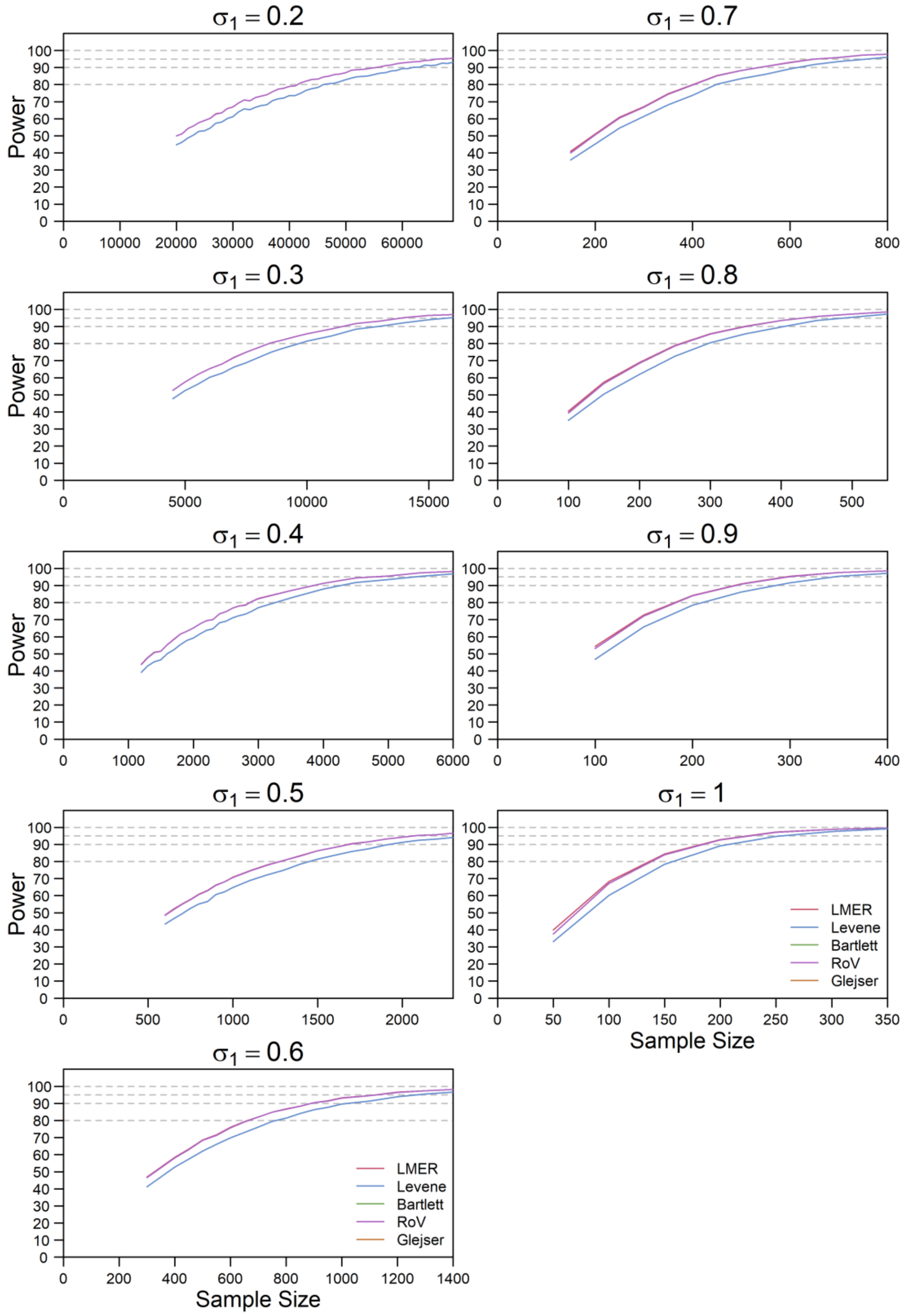

References

1. Cally JG, Stuart-Fox D, Holman L. Meta-analytic evidence that sexual selection improves population fitness. *Nature Communications* 2019;**10**(1):2017.

2. Chamberlain R, Brunswick N, Siev J, McManus IC. Meta-analytic findings reveal lower means but higher variances in visuospatial ability in dyslexia. *British Journal of Psychology* 2018;**109**(4):897-916.

3. Munkholm K, Winkelbeiner S, Homan P. Individual response to antidepressants for depression in adults-a meta-analysis and simulation study. *PLOS ONE* 2020;**15**(8):e0237950.

4. O'Dea RE, Lagisz M, Jennions MD, Nakagawa S. Gender differences in individual variation in academic grades fail to fit expected patterns for STEM. *Nature Communications* 2018;**9**(1):3777.

5. Pillinger T, Osimo E, Brugger S, et al. A Meta-analysis of Immune Parameters, Variability, and Assessment of Modal Distribution in Psychosis and Test of the Immune Subgroup Hypothesis. *Schizophrenia Bulletin* 2018;**45**(5):1120-1133.

6. Plöderl M, Hengartner MP. What are the chances for personalised treatment with antidepressants? Detection of patient-by-treatment interaction with a variance ratio meta-analysis. *BMJ Open* 2019;**9**(12).

7. Prendergast LA, Staudte RG. Meta-analysis of ratios of sample variances. *Statistics in Medicine* 2016;**35**(11):1780-1799.

8. Senior A, Nakagawa S, Raubenheimer D, Simpson S, Noble D. Dietary restriction increases variability in longevity. *Biology Letters* 2017;**13**(3).

9. English S, Uller TJBl. Does early-life diet affect longevity? A meta-analysis across experimental studies. 2016;**12**(9):20160291.

10. Senior AM, Gosby AK, Lu J, Simpson SJ, Raubenheimer D. Meta-analysis of variance: an illustration comparing the effects of two dietary interventions on variability in weight. *Evolution, Medicine, and Public Health* 2016;**2016**(1):244-255.

11. Williamson PJ, Atkinson G, Batterham AM. Inter-individual differences in weight change following exercise interventions: a systematic review and meta-analysis of randomized controlled trials. *Obesity Reviews* 2018;**19**(7):960-975.

12. Winkelbeiner S, Leucht S, Kane JM, Homan P. Evaluation of Differences in Individual Treatment Response in Schizophrenia Spectrum Disorders: A Meta-analysis. *JAMA Psychiatry* 2019;**76**(10):1063–1073.

13. Glejser H. A New Test for Heteroskedasticity. *Journal of the American Statistical Association* 1969;**64**(325):316-323.

14. Levene H. Robust Tests for Equality of Variances. In: Olkin I, ed. *Contributions to Probability and Statistics*. Palo Alto: Stanford Univ. Press, 1960.

15. Brown MB, Forsythe AB. Robust tests for the equality of variances. *Journal of the American Statistical Association* 1974;**69**(346):364-367.

16. Soave D, Sun LJB. A generalized Levene's scale test for variance heterogeneity in the presence of sample correlation and group uncertainty. 2017;**73**(3):960-971.

17. Bartlett MS. Properties of Sufficiency and Statistical Tests. *Proceedings of the Royal Society of London. Series A, Mathematical and Physical Sciences* 1937;**160**(901):268-282.

18. Harding B, Tremblay C, Cousineau D. Standard errors: A review and evaluation of standard error estimators using Monte Carlo simulations. *The Quantitative Methods for Psychology* 2014;**10**(2):107-123.

19. Nakagawa S, Poulin R, Mengersen K, et al. Meta-analysis of variation: ecological and evolutionary applications and beyond. *Methods in Ecology and Evolution* 2015;**6**(2):143-152.

20. Feltz CJ, Miller GE. An asymptotic test for the equality of coefficients of variation from k populations. *Statistics in Medicine* 1996;**15**(6):647-658.

21. Kessler D, Lewis G, Kaur S, et al. Therapist-delivered Internet psychotherapy for depression in primary care: a randomised controlled trial. *The Lancet* 2009;**374**(9690):628-634.

22. Koenker R. A note on studentizing a test for heteroscedasticity. *Journal of Econometrics* 1981;**17**(1):107-112.

23. Richards D, Richardson T. Computer-based psychological treatments for depression: a systematic review and meta-analysis. *Clinical Psychology Review* 2012;**32**(4):329-342.

24. Palmer SC, Navaneethan SD, Craig JC, et al. HMG CoA reductase inhibitors (statins) for people with chronic kidney disease not requiring dialysis. *Cochrane Database of Systematic Reviews* 2014(5).
